# Education level may modify the association between cardiac index and cognitive function among elders with normal ejection function

**DOI:** 10.3389/fcvm.2022.844396

**Published:** 2022-09-12

**Authors:** Hao-Min Cheng, Shao-Yuan Chuang, Yu-Ting Ko, Chao-Feng Liao, Wen-Harn Pan, Wen-Ling Liu, Chen-Ying Hung, Chen-Huan Chen

**Affiliations:** ^1^Department of Medical Education, Taipei Veterans General Hospital, Taipei, Taiwan; ^2^Faculty of Medicine, National Yang Ming Chiao Tung University School of Medicine, Taipei, Taiwan; ^3^Public Health Sciences Institute, National Health Research Institutes, Zhunan, Taiwan; ^4^Institutes of Biomedical Sciences, Academia Sinica, Taipei, Taiwan; ^5^Department of Internal Medicine, Taipei General Veterans Hospital, Hsinchu, Taiwan

**Keywords:** education, cognitive function, cardiac index, elderly population, cohort

## Abstract

**Background:**

Lower cardiac index (CI) in elders has been associated with incident dementia, and higher CI has protectively effect with brain aging. In the present study, we investigated the modulating effects of education level and arterial stiffness on the association between CI and cognitive function among older adults.

**Methods:**

A total of 723 elders (≥60 years, 50.1% women) with normal left ventricular ejection fraction (≥50%) were identified from the Cardiovascular Diseases Risk Factor Two-Township Study. CI was calculated from the Doppler-derived stroke volume. We evaluated arterial stiffness by measuring carotid-femoral pulse wave velocity (CFPWV) and global cognitive function by using the Mini-Mental Short Examination (MMSE). Education level was determined by years of formal education.

**Results:**

In linear regression analysis adjusting for age, sex, formal years of education, and CFPWV, CI was significantly positively associated with MMSE (BETA=0.344±0.130, *P* = 0.0082). In logistic regression analysis adjusting for age, sex, formal years of education, and CFPWV, subjects with a CI≥75 percentile had a significantly lower risk of low MMSE (<26) (OR = 0.495, 95% CI = 0.274–0.896, *P* = 0.02). In subgroup analysis, higher CI was significantly associated with higher MMSE and lower risk of low MMSE only in elders with ≤ 9 years of formal education. Causal mediation analysis suggests that higher CI maintains higher MMSE in elders with lower education levels whereas higher CFPWV causes lower MMSE in all the elders.

**Conclusion:**

In elders with normal ejection fraction, a higher CI was associated with a lower risk of cognitive function impairment, independent of arterial stiffness, mainly in subjects with a lower education level and possibly a smaller cognitive reserve.

## Background

Reduced systemic blood flow may reduce cerebral perfusion and cause subclinical brain injury, and thereby compromise cognitive function (1, 2). In the extreme, patients with systolic heart failure have a higher prevalence of cognitive impairment than those with normal cardiac function (3–5). In the community-based participants free from clinical stroke, transient ischemic attack, dementia, or heart disease, a subtle reduction in cardiac index (CI) may be associated with reduced brain volumes (1, 6), furthermore, the higher CI (top tertile) had a higher mean total brain volume equivalent to nearly brain aging compared with those participants in either the middle or bottom teriles of CI. Therefore, a higher CI, implying better systemic blood flow, may help ensure adequate cerebral blood flow (7) to prevent cognitive function decline due to aging.

In addition to aging, low education is a recognized risk factor for dementia (8). A higher level education in early life is usually associated with a significant reduction in prevalence and incidence of dementia (8). Education may influence the course and outcome of cognitive decline and protect against the onset of dementia, the so-called cognitive reserve hypothesis (8–11). The interaction between the effect of education on cognitive reserve and the joint effect of cardiac and arterial aging on cognitive decline remains poorly understood (12).

We hypothesized that the potential protective effect of relatively higher CI and the well-documented detrimental effect from arterial aging on cognitive decline may differ in elders with different education levels, because of the difference in cognitive reserve. Therefore, the present study aimed to elucidate the interrelationship of CI, arterial aging and education level with cognitive function among the elders with normal left ventricular ejection fraction in the community. Specifically, we investigated the modulating effects of education level and arterial stiffness on the association between CI and cognitive function among older adults.

## Methods

### Study population

The Cardiovascular Disease Risk Factors Two-Township Study (CVDFACTS) is an ongoing longitudinal study of the risk factors for and pathogenesis of cardiovascular disease in two Taiwanese townships, Chu-Dung (a Hakka community) and Pu-Tzu (a Fukienese community) (13). The CVDFACTS study was instituted in 1989–1991 (baseline) and had 4 waves of surveys (1991–1993, 1993–1997, 1997–1999, and 1999–2002). Due to carotid hemodynamic parameters were measured in the 4th wave, we invited those (*n* = 2,014) with carotid hemodynamic measures and aged more than 60 years old after January 2014, by letter or by telephone to attend the present study project entitled “The Impact of Pulsatile Hemodynamics on Elderly Cognitive Function: the Cardio-cerebral Interactions” conducted between 2014 and 2016. Those none participants had higher age (77 vs. 69 years old, *p* < 0.05), lower male proportion (43 vs. 50%, *p* < 0.05) and shorter schooling education (7 vs. 10 years, *p* < 0.05), compared to the participants. The study protocol was approved by the Institutional Review Board of National Yang-Ming University. Each participant was well informed, and a written consent was obtained before entering the study.

In total, two visits of data collection within 3 months were arranged for each participant. In the first visit, personal characteristics, anthropometric measurements, cognitive function assessment, and fasting blood samples were collected. The histories of stroke and heart disease were collected by the structured questionnaires such as: “Did you have heart disease diagnosed by a physician at a clinic or hospital?”. The second visit involved the measurements of cardiovascular hemodynamics.

**Figure 1:**
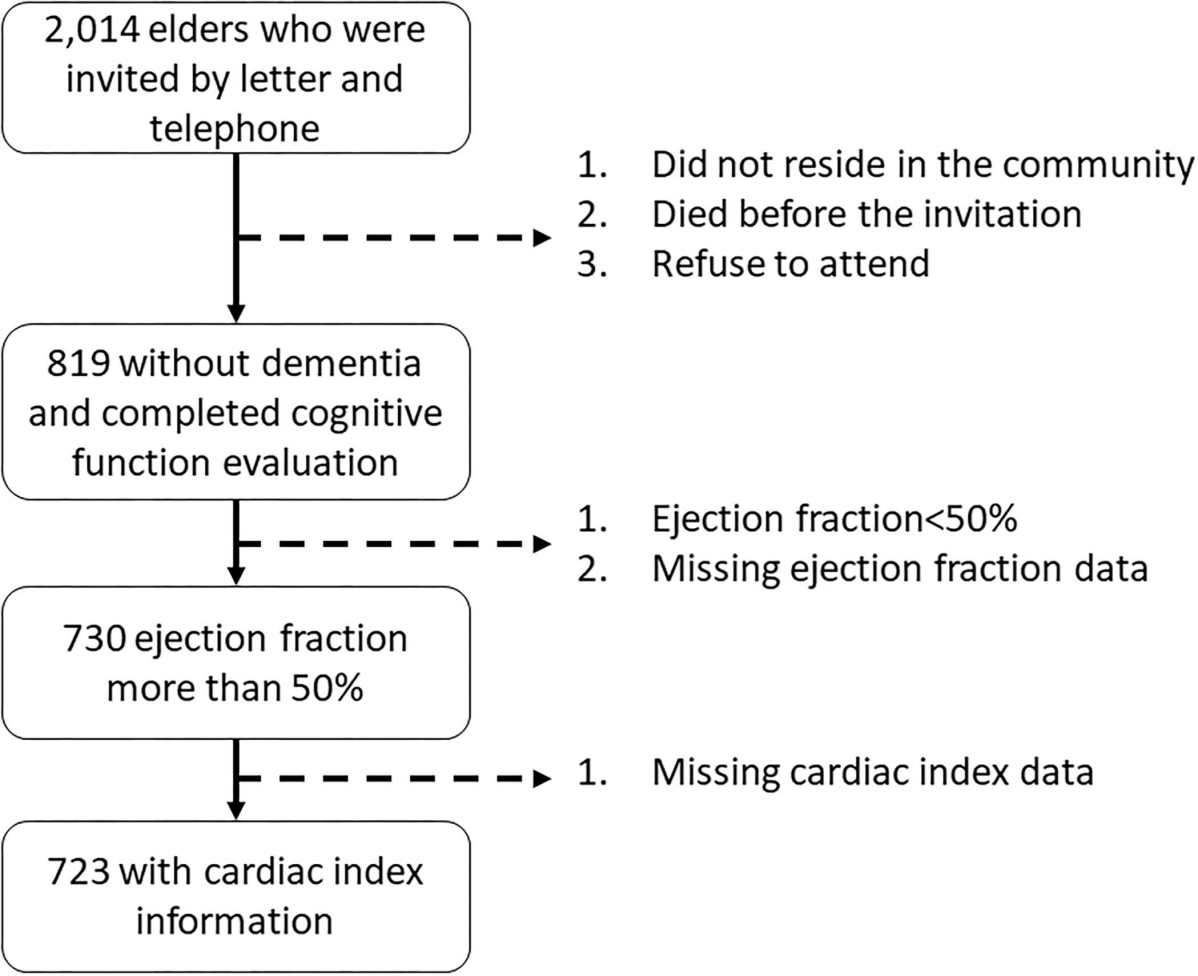
Study population flowchart.

A total of 819 elders aged 60 years or more participated in the project and completed the cognitive function assessment. For the purpose of the present analysis, we excluded 89 subjects with a left-ventricular ejection fraction <50% or missing ejection fraction data, and 7 additional subjects with missing CI data. The cognitive function had no difference between elders with and without ejection fraction <50%. Finally, 723 subjects aged ≥ 60 years and with normal left ventricular ejection fraction (>50%) were eligible and included in the present analysis (Figure 1).

### Measurements

#### Cognitive function

The global cognitive function was assessed using the Mini-Mental Short Examination (MMSE), (14). Chinese version for Taiwan, *via* face-to-face interview with the well-trained study nurses at the study sites. The MMSE consists of 20 items clustered into 11 subscores to assess different aspects of cognitive function, namely, orientation, memory, attention, calculation and following a three-stage command, and has a total score of 30 points (14). A cut-off point of 26 was used to define cognitive impairment.

#### Education level

The usual formal education in Taiwan is elementary school (6 years), junior high school (3 years), senior high school (3 years), college (4 years), and graduate school (2–4 years). Total years of formal education were registered for each participant and a cut-off point 9 years was used to define lower education level ( ≤ 9 years), since 9-year education was cumulated school years of elementary and junior high school.

#### Cardiac index

All the subjects received a transthoracic echocardiography performed by the same experienced sonographer using a commercially available machine (HD11 XE Ultrasound system, Koninklijke Philips N.V.). Therefore, there was no intra-measurers errors in this study. All the images were digitized for off-line analysis by the sonographer, using the TomTec Image-Arena™ Software 4.0 (TomTec Imaging Systems GmbH, Munich, Germany). Left ventricular volume was measured from the summation of a stack of elliptical disks by tracing the endocardial border of the left ventricle at end-diastole and end-systole in apical 4 chamber view. Left ventricular ejection fraction was calculated from the M-mode measurements. Doppler-derived stroke volume was the product of the cross-sectional area of the left ventricular outflow tract and the flow across the left ventricular outflow tract, which is determined by the velocity time integral of the Doppler signal during systole (15). Doppler-derived cardiac output was calculated as stroke volume times heart rate and CI was cardiac output divided by body surface area (15).

#### Arterial stiffness

Applanation tonometry was performed with a pencil-type tonometer incorporating a high-fidelity strain-gauge transducer in a 7-mm-diameter flat tip (SPC-350, Millar Instruments Inc, Texas) to record the pulse waveforms at the right common carotid artery and right femoral artery sequentially (16). CFPWV was estimated by the distance between the right carotid and right femoral artery measured by a measuring tape divided by the pulse transit time. The pulse transit time between the right carotid artery and the right femoral artery was calculated by a simultaneously recorded ECG signal using a custom-designed software on a commercial software package (Matlab, version 4.2, The MathWorks, Inc.) (16).

#### Others

Supine brachial systolic and diastolic blood pressure were measured at the right arm using automated analyzer, VP-1000 (Colin Co., Komaki, Japan) with an appropriate-sized cuff at heart level. Body surface area was calculated by the product of body height (cm) and body weight (kg) divided by 3,600. Body mass index was estimated by the body weight in kg divided by body height in meter.

### Statistical analysis

Characteristics of study population and subgroups of higher and lower education levels > and ≤ 9 years of formal education) were presented as mean and SD for interval variables, and number with proportion for categorical variables. The Students *t*-test was used to compare the mean difference between two groups and appropriate *p*-value with fitting assumptions or not was presented.

Association of MMSE with CI and CFPWV was evaluated by univariable and multivariable linear regression analyses for the total study population and subgroups of education levels. Association between low MMSE (total score <26) and quartile analyses of CI and CFPWV was evaluated by multivariable logistic regression analyses for the total population and subgroups of education levels. We also evaluated the modulating effect of education on the association between cardiac index and cognitive function by stratified analysis and interaction evaluation. The estimated minima sample size for the study hypothesis that cardiac index associated with MMSE score (*r* = 0.30) was 195 for 0.05 alpha value with two-tail, power with 99%.

We further constructed causal models to elucidate the significance of CI and CFPWV as mediators on the causal pathway between advancing age and declining MMSE, with years of formal education as a confounder. The path analysis was frequently used to explore the potential cause relationship among a set of observed variables. The process CALIS in the statistic software SAS was usually conducted for path analysis. The direct effect of each pathway in the causal models was estimated by a Path coefficient and its *P*-value and the goodness-of-fitness index of each model was presented. All the statistical analysis were conducted in SAS 9.4. Significance level was set at 0.05.

## Results

### Characteristics of study population

Among the 723 eligible elders with normal left ventricular ejection fraction (mean age 69.2 ± 7.2 years, 49.93% women, average left ventricular ejection fraction 71.3 ± 6.7%), the average MMSE score was 27.9 ± 2.7 (28.3 ± 2.07 for men, 27.6 ± 3.08 for women, P = 0.0008), and 97 subjects had an MMSE <26 (13.4%; 10.8% for men vs. 16.0% for women, *P* = 0.0395) (Table 1).

**Table 1:** Characteristics of the study population with lower and higher levels of education (*n* = 723).

**Variable**	**Total (*n =* 723)**	**Lower education level ( ≤ 9 years) (*n =* 231)**	**Higher education level (>9 years) (*n =* 492)**	**P value**
Age, years	69.2 ± 7.2	72.3 ± 7.6	67.8 ± 6.5	<0.0001
Male gender, n (%)	361 (49.9)	86 (36.9)	275 (55.9)	<0.0001
Formal education, years	10.3 ± 4.2	5.2 ± 1.9	12.6 ± 2.7	<0.0001
Body mass index, kg/m^2^	24.7 ± 3.4	25.3 ± 3.3	24.4 ± 3.4	0.0013
Brachial systolic BP	133.4 ± 17.7	136.3 ± 17.3	132.0 ± 17.8	0.0024
Brachial diastolic BP	77.1 ± 10.3	77.2 ± 9.3	77.0 ± 10.7	0.8188
Triglycerides, mg/dL	127.4 ± 77.5	134.1 ± 77.1	124.1 ± 77.4	0.1066
HDL-cholesterol, mg/dL	54.3 ± 15.6	53.3 ± 14.1	54.8 ± 16.3	0.2293
LDL-cholesterol, mg/dL	116.3 ± 34.6	116.7 ± 34.2	116.4 ± 35.0	0.9114
Total cholesterol, mg/dL	195.8 ± 39.5	196.5 ± 38.5	195.6 ± 40.0	0.7766
Fasting glucose, mg/dL	104.3 ± 26.3	107.6 ± 31.3	102.7 ± 23.4	0.0192
Cardiac index, L/min/m^2^	2.8 ± 0.7	2.92 ± 0.75	2.75 ± 0.65	0.0022
CFPWV, m/sec	13.7 ± 4.6	14.6 ± 5.2	13.3 ± 4.3	0.0007
Ejection fraction, %	71.3 ± 6.7	71.3 ± 6.8	71.3 ± 6.7	0.9677
MMSE	27.9 ± 2.7	26.3 ± 4.3	28.6 ± 1.8	<0.0001
MMSE <26, n (%)	97 (13.4)	68 (29.2)	31 (6.3)	<0.0001
Heart disease, n (%)	151 (20.9)	59 (25.3)	92 (18.7)	0.0403
Stroke history, n (%)	24 (3.3)	12 (5.2)	12 (2.4)	0.0567

BP, blood pressure; CFPWV, carotid-femoral pulse wave velocity; HDL, high-density lipoprotein; LDL, low-density lipoprotein; MMSE, Mini-Mental State Examination.

Subjects with a lower education level ( ≤ 9 years of formal education, average 5.2 years) were significantly older, had a significantly lower MMSE score and higher prevalence of cognitive impairment (MMSE <26), and had a significantly higher CI and CFPWV than those with a higher education level (>9 years of formal education and average 12.6 years) (Table 1).

Subjects with a lower education level had a higher proportion of women, greater body mass index, higher brachial systolic blood pressure and fasting glucose, and a higher prevalence of heart disease, compared to those elders with a higher education level (Table 1).

### Association of MMSE with CI and CFPWV

In the multivariable analysis for the study population, CFPWV was significantly negatively, and CI was significantly positively associated with MMSE, when age, gender, and years of formal education were included in the model (Table 2). In subjects with a lower education level, CFPWV was significantly negatively, and CI was significantly positively associated with MMSE (Table 2). In contract, in subjects with a higher education level, CFPWV remained significantly negatively associated with MMSE, but CI was no longer associated with MMSE (Table 2).

**Table 2:** Association of MMSE with CI and CFPWV, adjusted for age, sex and education whole population and stratified by levels of education.

**Variable**	**Total (*n =* 723)**		**Lower education level ( ≤ 9 years) (*n =* 231)**		**Higher education level (>9 years) (*n =* 492)**	
**Multivariable analysis**	**BETA (SE)**	***P* value**	**BETA (SE)**	***P* value**	**BETA (SE)**	***P* value**
Age, years	−0.071 (0.014)	<0.0001	−0.052 (0.030)	0.089	−0.068 (0.013)	<0.0001
Gender, male vs. female	0.490 (0.186)	0.0086	0.737 (0.436)	0.0928	0.346 (0.165)	0.0362
Formal education, years	0.197 (0.023)	<0.0001	0.701 (0.110)	< .0001	0.013 (0.030)	0.6507
CFPWV, m/sec	−0.070 (0.021)	0.0008	−0.090 (0.042)	0.0341	−0.045 (0.020)	0.0250
CI, L/min/m^2^	0.344 (0.130)	0.0082	0.649 (0.274)	0.0187	0.093 (0.112)	0.4467

BETA, standardized regression coefficient; CFPWV, carotid-femoral pulse wave velocity; CI, cardiac index; SE, standard error of BETA.

### Association between cognitive impairment and quartile analyses of CI and CFPWV

In subjects with a lower education level, subjects in the upper quartile of CI, whereas subjects in the upper quartile of CFPWV were not significantly associated with a higher risk for cognitive impairment (*P* = 0.0592). In contrast, in subjects with a higher education level, subjects in the upper quartile of CFPWV were significantly associated with a higher risk, whereas subjects in the upper quartile of CI were not associated with a lower risk of cognitive impairment (Table 3, separate upper quartile analysis).

**Table 3:** Association between low MMSE and cardiac index, carotid-femoral pulse wave velocity and education years, multivariable logistic analyses stratified by levels of education.

**Variable**	**Total (*n =* 723)**		**Lower education level (<9 years) (*n =* 231)**		**Higher education level (≥9 years) (*n =* 492)**	
	**OR (95% CI)**	**P value**	**OR (95% CI)**	**P value**	**OR (95% CI)**	**P value**
**Bivariate upper quartile analysis**						
Cardiac index, ≥ vs. <75th percentile	0.495 (0.274–0.896)	0.0202	0.357 (0.158–0.808)	0.0134	0.788 (0.314–1.977)	0.6116
CFPWV, ≥ vs. <75th percentile	2.187 (1.287–3.716)	0.0038	1.947 (0.939–4.037)	0.0732	2.553 (1.100–5.925)	0.0292
**Combined upper quartile analysis**						
Higher CI (≥75^th^ percentile) and lower CFPWV(<75^th^ percentile) (*N =* 133)	0.246 (0.112–0.542)	0.0005	0.211 (0.073–0.609)	0.0040	0.297 (0.077–1.149)	0.0786
Lower CI (<75^th^ percentile) and lower CFPWV(<75^th^ percentile) (*N =* 409)	0.403 (0.221–0.737)	0.0031	0.385 (0.167–0.889)	0.0040	0.406 (0.156–1.057)	0.0649
Higher CI (≥75^th^ percentile) and higher CFPWV(≥75^th^ percentile) (*N =* 49)	0.364 (0.143–0.929)	0.0345	0.171 (0.043–0.671)	0.0254	0.845 (0.238–3.001)	0.7940
Lower CI (<75^th^ percentile) and higher CFPWV (≥75^th^ percentile) (*N =* 132)	1.0 (referent)		1.0 (referent)		1.0 (referent)	

All the models were adjusted for age, sex, and years of formal education.

CI, cardiac index; CFPWV, carotid-femoral pulse wave velocity. All the subjects were then divided into 4 subgroups according to higher and lower CI and CFPWV using the sex-specific 75^th^ percentile as the cut-points.

In the logistic regression model, upper quartile CI and upper quartile CFPWV were negatively and positively significantly associated with cognitive impairment, respectively (Table 3, bivariate upper quartile analysis). In subjects with a lower education level, upper quartile CI was significantly associated with a lower risk, and upper quartile CFPWV was not significantly associated with a higher risk for cognitive impairment (*P* = 0.0732). In contrast, in subjects with a higher education level, upper quartile CFPWV was significantly associated with a higher risk, whereas upper quartile CI was not associated with a lower risk for cognitive impairment (Table 3, bivariate upper quartile analysis).

In total, four subgroups were generated according to higher and lower CI and CFPWV. The subjects in the subgroups of higher CI and lower CFPWV, higher CI and higher CFPWV, and lower CI and lower CFPWV had significantly lower risks of cognitive impairment as compared with the referent subgroup of lower CI and higher CFPWV when age, sex, and years of formal education were accounted for (Table 3, combined upper quartile analysis). Similar significant results were observed in subjects with a lower education level but not in those with a higher education level.

### Modulating effects of education on the relationship between cardiac index and MMSE

We conducted a modulate analysis to evaluate the modulating effect of education on the association between CI and MMSE. The interaction variable of education and CI for MMSE was borderline significant with *p* = 0.07. This result indicates that the association between cardiac index and cognitive function was slightly modulated by education, but not reach statistic significant.

**Figure 2:**
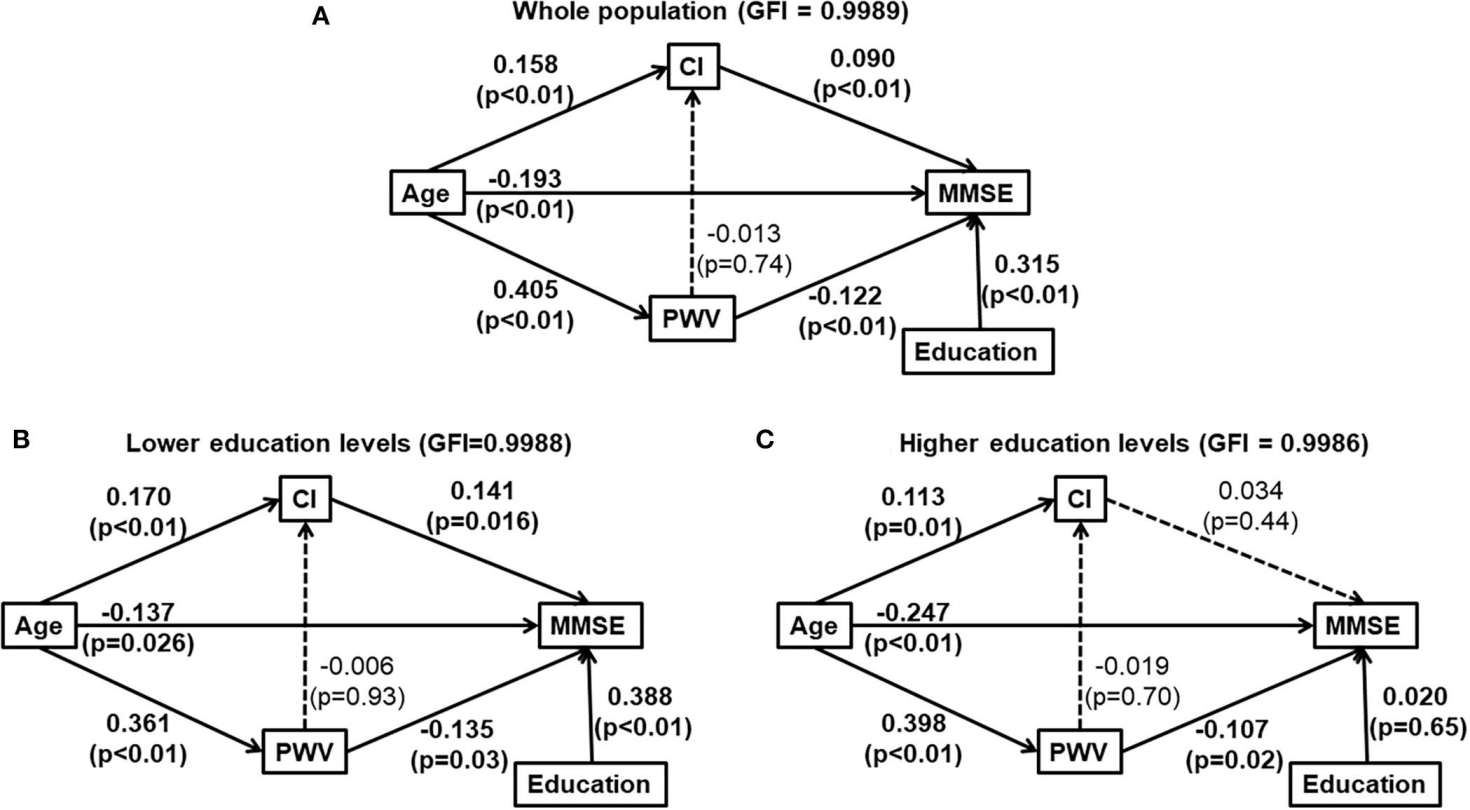
Path analysis diagrams for the whole study population **(A)**, subjects with lower education levels [ ≤ 9 years of formal education, **(B)**] and those with higher education levels [>9 years of formal education, **(C)**]. Solid line indicates significant association. Dotted line indicates none-significant association. Path coefficient and its *P*-value are presented for the evaluation of direct effect between two adjacent variables. **(A)** was for whole population and **(B,C)** were for those elders with and without lower education, respectively. CI, cardiac index; education, years of formal education; GFI, the goodness of fitness index; MMSE, Mini-Mental State Examination; PWV, carotid-femoral pulse wave velocity; The interrelationship of cardiac index (CI), carotid-formal pulse wave velocity (PWV), education and cognitive function (Mini-Mental State Examination).

We further conducted path analysis to describe the potential causal interrelationships of age, cardiac index, carotid-femoral pulse wave velocity, education, and cognitive function. According to the Path analysis with years of formal education as a confounder, advancing age might directly decrease MMSE, and indirectly affect MMSE favorably and unfavorable by increasing CI and CFPWV for the total study population (Figure 2A, and in subjects with a lower education level (Figure 2B). In contrast, in subjects with a higher education level, advancing age might directly decrease MMSE and indirectly decrease MMSE through increased CFPWV. However, the favorable effect of increased CI on MMSE was no longer observed (Figure 2C).

## Discussion

### Main findings

Our study found that education level could modify the association between CI and cognitive function among elders with normal left ventricular ejection. The association between cardiac index and MMSE score was significant only in elders with a lower education level but was not significant in those with a higher education level. Furthermore, causal inference by the Path analysis also supports that higher CI maintains higher MMSE in elders with the lower education levels. Thus, elders with a lower education level may have a lower cognitive reserve and may be more vulnerable to the adverse effect of a subtle reduction of systemic blood flow on cognitive function.

Our study also found that CI and CFPWV simultaneously and independently contribute to the pathogenesis of cognitive function decline in the elders, especially in those with a lower education level, strategies to preserve systemic blood flow and prevent arterial stiffening may be considered for maintaining or restoring brain health. Enrichment of cognitive reserve through early life education and life time learning may help preserve cognitive function during late life.

### Cardiac index and cognitive function in other studies

Few studies investigated the relationship between cardiac function and cognitive function among general population with normal cardiac function (1, 17, 18). The Framingham Offspring Cohort participants free of clinical stroke, transient ischemic attack, dementia, and clinically prevalent cardiovascular disease, CI was significantly positively related to total brain volume, and low CI was related to poorer performances on information processing/executive function with borderline significance (1). Follow-up of the same cohort excluding clinically prevalent cardiovascular disease and atrial fibrillation revealed that individuals with clinically low CI (<2.5 L/min/m^2^) had a higher relative risk of both dementia and Alzheimer's disease compared with individuals with normal CI (19).

However, the underlying mechanisms of the association between cardiac function and cognitive function among subjects with normal cardiac function were unclear, and the mechanisms underlying clinically low CI in about one-third of the ambulatory older adults were unknown (19).

Our study results may compliment the Framingham Offspring Cohort study, which showed the harmful effect of a clinically significant low CI, by clearly demonstrating the protective effect of a higher CI (upper quartile) in preventing the age-related cognitive function decline, independent of age-related arterial stiffening. Overall, our results may suggest that a normal systemic blood flow is important in maintaining adequate cerebral blood flow and normal cognitive function, especially in elders with a lower cognitive reserve.

The association between cardiac index and cognitive function decline maybe inked by cerebral blood flow. Cerebral hypoperfusion resulting from the reduced systemic perfusion has been considered as the major cause of cognitive function impairment in heart failure patients (3–5). Cerebral blood flow is substantially reduced in patients with severe heart failure and it may be reversible after heart transplantation (5). Moreover, it has been shown that decreased CI was associated with smaller subcortical gray matter volume in patients with heart failure. Furthermore, cardiac resynchronization therapy in the moderate-to-severe heart failure patients may improve left ventricular ejection fraction and enhance cognitive outcome, namely, global cognition, executive function, and visuospatial function (20).

### Modulating effect of cognitive reserve on cognitive function decline

Cognitive reserve refers to the ability of the brain to optimize or maximize performance through differential recruitment of brain networks or use of alternative strategies against brain damage (11). Subjects with a higher education level usually experience less cognitive changes in the presence of age-related or Alzheimer's disease, probably because of a higher cognitive reserve (21). High education in early life may help to postpone cognitive and brain reserve decline in normal aging (22). Cognitive reserve can be measured by proxy indicators, such as years of full-time education and occupational complexity (23). The Cognitive Function and Aging Study Wales cohort reported cognitive reserve was an important mediator of the association between lifestyle factors and cognitive function, with indirect effects *via* cognitive reserve contributing 21% of the overall effect on cognition (23). In our study, education level measured by years of formal education was significantly associated with MMSE in both univariable and multivariable linear regression analyses (Table 2). In Path analysis, a formal education year was significantly associated with a higher MMSE score in subjects with a lower education level, but not in those with a higher education level (Figures 2B,C). Moreover, the protective effect of higher CI on preserving MMSE was also significant only in subjects with a lower education level. These results may support that cognitive reserve plays an important role in the development of cognitive dysfunction in later life. Elders with a higher education level, implying the presence of a higher cognitive reserve, may rely less on the protective effect from a higher CI.

### Artery stiffness and cognitive function decline

It has been shown that marked stiffening of the aorta may augment the transmission of excessive flow pulsatility into the brain, causing microvascular structural brain damage and various cognitive function impairments (24). Our study also found that a higher CFPWV was significantly and independently associated with a lower MMSE. Moreover, we found that the effects of CFPWV and CI on MMSE were additive in elders with a lower education level but not in those with a higher education level. In Path analysis, age had a significant direct effect and two separate and independent significant indirect effects *via* CI and CFPWV, respectively, on MMSE. The indirect effect *via* CFPWV was significant in elders regardless of their education levels. In contrast, the indirect effect *via* CI was significant only in elders with a lower education level. These results may suggest that higher cognitive reserve does not prevent the age-related arterial stiffness, or arterial aging, from damaging the brain (3). Strategies to slow down or reverse arterial aging may be needed to maintain brain health.

### Limitations and strength

Several limitations in this study are addressed as follows. First, this study was a cross-sectional design and therefore the results do not prove the causal inference. In the current design, we did not avoid the possible reverse causality which a small brain requires a small Cardiac index. This issue needs further investigation. Second, the modulating effect of education levels on the association between CI and cognitive function observed in our study may not be extrapolated to other populations that have a higher homogeneity in education levels. Third, we did not have the reliability information of hemodynamic parameters, however, all hemodynamic parameters were performed by a well-trained technician to avoid the intra measurer error.

## Conclusion/Implication

In elders with normal ejection fraction, a higher CI was associated with a lower risk of cognitive function impairment, independent of arterial stiffness, mainly in subjects with a lower education level and possibly a smaller cognitive reserve. A higher education level may imply a higher cognitive reserve that may not require the protective effect of high CI on cognitive function.

## Data availability statement

The original contributions presented in the study are included in the article/supplementary material, further inquiries can be directed to the corresponding author.

## Ethics statement

The studies involving human participants were reviewed and approved by the Institutional Review Board of National Yang-Ming University. The patients/participants provided their written informed consent to participate in this study. Written informed consent was obtained from the individual(s) for the publication of any potentially identifiable images or data included in this article.

## Author contributions

Manuscript drafting and study design: H-MC, S-YC, C-HC, and W-HP. Data-analysis: S-YC and W-LL. Data collection and bio-information capture: Y-TK, C-YH, and C-FL. All authors contributed to the article and approved the submitted version.

## Funding

This study was supported by Ministry of Science and Technology (MOST-103-2314-B-400-007, MOST-104-2314-B-400-019, MOST-105-2314-B-400-004, MOST-105-2314-B-010-001, MOST-108-2314-B-010-001, MOST-110-2321-B-400-004, and MOST-111-2321-B-400-004) and an intramural grant from National Yang-Ming Chiao Tung University (E107F-M01-0501).

## Conflict of interest

The authors declare that the research was conducted in the absence of any commercial or financial relationships that could be construed as a potential conflict of interest.

## Publisher's note

All claims expressed in this article are solely those of the authors and do not necessarily represent those of their affiliated organizations, or those of the publisher, the editors and the reviewers. Any product that may be evaluated in this article, or claim that may be made by its manufacturer, is not guaranteed or endorsed by the publisher.

## Abbreviations

CI: Cardiac Index
CFPWV: Carotid-Femoral Pulse Wave Velocity
MMSE: Mini-Mental Short Examination
CVDFACTS: The Cardiovascular Disease Risk Factors Two-Township Study.
